# Change of the Neutrophil-to-Lymphocyte Ratio during Treatment: A Potential Prognostic Biomarker in Metastatic Prostate Cancer Treated with Radium-223 Dichloride

**DOI:** 10.3390/cancers14194606

**Published:** 2022-09-22

**Authors:** Kevin Kaulanjan, Johanna Dahan, Cédric Charrois-Durand, Fred Saad, Laurent Brureau, Guila Delouya, Daniel Taussky, Edouard Auclin

**Affiliations:** 1 Institut du Cancer de Montréal (ICM), Centre de Recherche du Centre Hospitalier de l’Université de Montréal (CRCHUM), Montreal, QC H2X 0A9, Canada; 2 Department of Radiation Oncology, Centre Hospitalier de l’Université de Montréal (CHUM), Montreal, QC H2X 3E4, Canada; 3 Department of Surgery, Division of Urology, Centre Hospitalier de l’Université de Montréal (CHUM), Montreal, QC H2X 3E4, Canada; 4 Department of Urology, CHU de Guadeloupe, 97110 Pointe-à-Pitre, France

**Keywords:** metastatic prostate cancer, Radium-223, neutrophil to lymphocyte ratio, prognostic factor

## Abstract

**Simple Summary:**

We analyzed the influence of the neutrophil to lymphocyte ratio (NLR) and its change during therapy as a potential prognostic marker in metastatic prostate cancer patients treated with ^223^Radium (^223^Ra) and patients treated with docetaxel. We found that a low NLR at baseline as well as at 12 weeks of treatment was associated with a better overall survival only in patients treated with ^223^Ra but not in patients treated with Docetaxel. Patients with a baseline NLR ≤ 5 that remained low (NLR ≤ 5) at 12 weeks of treatment had significantly longer median survivals compared to patients whose NLR was low (<5) at baseline and that converted at 12 weeks to >5. The prognostic value of NLR at baseline and 12 weeks will need to be validated in larger prospective cohorts.

**Abstract:**

The neutrophil to lymphocyte ratio (NLR) at baseline has been shown to have prognostic value in metastatic prostate cancer. Little is known about the importance of a change in the NLR during treatment in patients treated with Radium-223 (^223^Ra). We investigated the prognostic value of the NLR at baseline and during therapy in patients with metastatic prostate cancer treated with ^223^Ra and also in patients treated with Docetaxel. We reviewed all patients treated with ^223^Ra in our center and randomly chosen patients treated with Docetaxel. Patients were stratified according to NLR ≤ 5 and >5 at baseline and at 12 weeks of therapy. The relationship between NLR measured at baseline and at 12 weeks and overall survival (OS) were evaluated. A total of 149 patients treated with ^223^Ra and 170 with Docetaxel were evaluated. For patients treated with ^223^Ra, overall survival was significantly better in patients that had both an NLR ≤ 5 at baseline and at 12 weeks. No such effect of NLR was found in patients treated with Docetaxel. In the present study, NLR at baseline and after 12 weeks of therapy was found to be prognostic factor in patients treated with ^223^Ra but not in those treated with Docetaxel.

## 1. Introduction

Prostate cancer (PCa) is the most commonly diagnosed cancer in men in the western world and the fifth leading cause of death worldwide [1]. In metastatic prostate cancer, especially in metastatic castration resistant prostate cancer (mCRPC), several life-extending therapeutic options are now available, such as androgen-signaling-targeted inhibitors, chemotherapy, and Radium-223 [2]. Despite, the therapeutic progress made recently, life expectancy remains poor. A better understanding of the predicting treatment response is needed to optimize the use and the sequencing of available therapies [3]. Lymphocytes and neutrophils have been implicated in host immune response to cancer including prostate cancer, either as a factor of host defense or tolerance [4]. Independently, the lymphocyte and neutrophil count depends on many factors and can be influenced by systemic therapy such as chemotherapy or immunotherapy. The neutrophil to lymphocyte ratio (NLR) is thought to reflect systemic inflammation and host response. The NLR, a simple measure of the absolute neutrophil count (ANC) divided by the absolute lymphocyte count (ALC) has been shown to be such a prognostic factor in many cancers as well as in urological cancers including prostate cancer [5,6]. NLR at baseline had a significant prognostic value in oncological outcomes [7,8]. Moreover, recent studies showed in kidney and lung cancers that a change of the NLR from a favorable ratio at baseline to an unfavorable ratio during treatment with immunotherapy was a negative prognostic factor of overall survival [9,10].

The bone selective, calcium mimetic Radium-223 dichloride (^223^Ra) was approved in mCRPC after docetaxel administration or if docetaxel is contraindicated [11]. It is a short-range, alpha particle emitter that might influence antigen presentation and could have a direct effect on the bone marrow. One could therefore expect a direct influence of ^223^Ra on the NLR while cytotoxic chemotherapy with docetaxel may have a different effect on the lymphocytes and neutrophil count [12].

The aim of the study was to analyze the influence of NLR and its change during therapy and its prognostic value in patients with metastatic prostate cancer treated with ^223^Ra and patients treated with docetaxel.

## 2. Materials and Methods

### 2.1. Study Population

All patients with a metastatic castration-resistant prostate cancer treated with ^223^Ra at Centre Hospitalier de l’Université de Montréal (CHUM) between October 2013 and March 2022 were included. Inclusion criteria were adult patients with histologically proven metastatic prostate cancer. As recommended by provincial guidelines, Radium-223 was offered to patients with castration-resistant prostate cancer with symptomatic bone metastases if they had been treated priorly with docetaxel or if docetaxel was contraindicated or not suitable for them. Patients treated with Docetaxel were randomly chosen among the large number of patients treated in our institution. Docetaxel was administered for either de novo metastatic castration-sensitive or metastatic castration-resistant prostate cancer. Patients were excluded if they had less than 3 cycles of ^223^Ra and less than 4 cycles of Docetaxel. Eligible patients were included in our institutional database. The current study was conducted according to the Declaration of Helsinki and approved by the institutional ethics committee’s (CER 21.328).

### 2.2. Study Variables and Outcomes

At baseline, patients’ age, Eastern Cooperative Group (ECOG) performance status, prostate specific antigen (PSA) level, hemoglobin level (Hb), full blood count (including absolute neutrophil and lymphocyte counts), and total alkaline phosphatase (tALP) levels were obtained. Before each cycle of ^223^Ra and docetaxel, all laboratory values were repeated. The neutrophil-to-lymphocyte ratio (NLR) was calculated as the ratio of the absolute neutrophil count (ANC) divided by the absolute lymphocyte count (ALC). We measured the baseline values and the values before the 3rd cycle of ^223^Ra and 4th cycle of Docetaxel, corresponding in both cases to 12 weeks after the beginning of therapy. Patients were stratified according to NLR in low NLR (NLR ≤ 5) and high NLR (NLR > 5). This cut-off was chosen because it had been shown to have prognostic value in previous studies in melanoma and lung cancer [13,14].

The NLR was recalculated at 12 weeks. Combining the results of baseline and at 12 weeks, a NLR score was calculated. Patients were stratified in “good” score recorded as a patient who had both a baseline and 12 weeks NLR of ≤5, an “intermediate” score those with NLR ≤ 5 at baseline and >5 at 12 weeks. Furthermore, a “poor” score was given to patients that had NLR > 5 both at baseline and at 12 weeks of treatment. The primary endpoint was overall survival (OS) defined as the time between the treatment onset (^223^Ra or Docetaxel) and death from any cause.

### 2.3. Statistical Analysis

Descriptive statistics were reported as median and interquartile range (IQR) for continuous variables, and as frequencies and percentages for categorical variables. Median and proportions were compared by the chi-square test or Fisher exact test, depending on the type of variable. OS was estimated by the Kaplan–Meier method and compared with the Log Rank test. Follow-up was calculated using the reverse Kaplan–Meier method. The association of clinical and biological variables with OS was first estimated with univariate Cox proportional hazard models, then with multivariable Cox regression model if parameters had *p* < 0.10 or was clinically relevant. Statistical analyses were performed using R Version 3.4.3 (The R foundation, Vienna, Austria). All tests were two-sided at a level of significance of *p* < 0.05 was used.

## 3. Results

### 3.1. Descriptive Characteristics of the Study Population

A total of 319 patients were analyzed, 149 (46.71%) were treated with ^223^Ra and 170 (54.29%) with Docetaxel. Baseline characteristics of ^223^Ra and Docetaxel are presented in Table 1a,b. Median age was 72 years (interquartile range, IQR 65–79) and 67 (IQR 59–74) years in ^223^Ra-treated patients and Docetaxel-treated patients, respectively. The median follow-up was 68.1 (95% CI 20.4-Not reached) months in ^223^Ra-treated patient and 77.8 (95% CI 68.8-Not reached) months in docetaxel-treated patients. 

There was no significant difference between patients with a baseline NLR ≤ 5 or >5 for ^223^Ra and docetaxel regarding PSA, tALP, hemoglobin and platelets. However, in the NLR > 5 group patients treated with ^223^Ra had a significantly higher white blood count (7.7 (6.8; 9)) than the NLR ≤ 5 group (6.2 (5.2; 7.9)). This difference in white cell count was not seen in the Docetaxel group.

### 3.2. Primary Outcome within Radium-Treated Population

#### 3.2.1. Association of NLR at Baseline with Overall Survival

**Figure 1:**
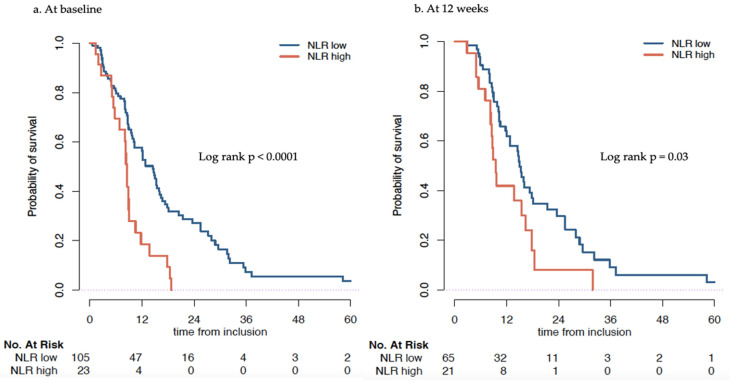
Overall survival (months) of patients treated with ^223^Ra estimated by Kaplan–Meier stratified by Neutrophil to Lymphocyte ratio (NLR) low (≤5) and NLR high (>5) calculated at baseline (**a**) and at 12 weeks (**b**).

In the ^223^Ra cohort, low NLR ≤ 5 at baseline was associated with better overall survival: 14.5 months (95% CI 10.2–16.4) vs. 8.5 months (95% CI 6.8–10.5) in the high NLR > 5 group, log rank *p* < 0.0001 (Figure 1a). In multivariable analysis, adjusted on prior docetaxel treatment, previous prostatectomy, ECOG, PSA level, Hemoglobin, tALP, NLR at baseline was an independent predictive factor (HR 1.7, 95% CI 1.00–2.90, *p* = 0.05) (Table 2, Supplementary Table S1).

#### 3.2.2. Association of Change of NLR at 12 Weeks with OS

**Figure 2:**
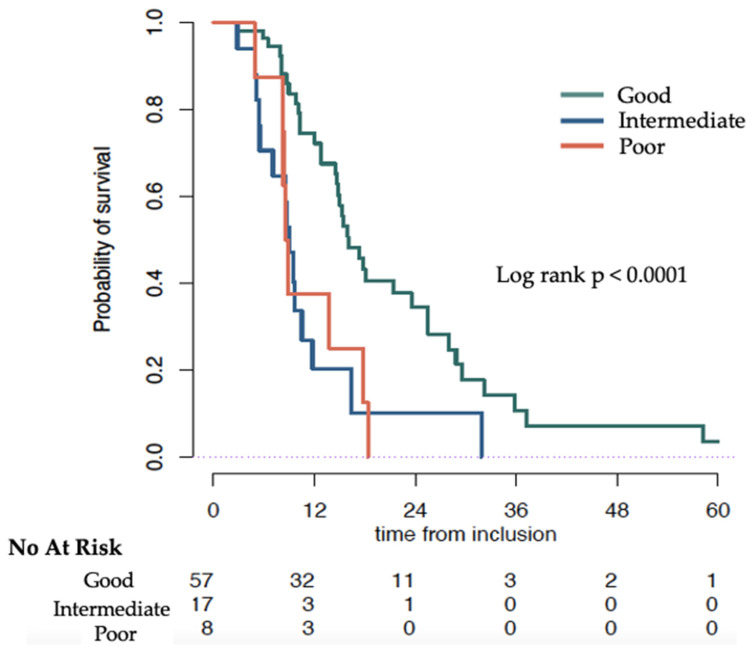
Overall survival (months) of patients treated with ^223^Ra estimated by Kaplan–Meier stratified by Neutrophil to Lymphocyte ratio (NLR) score: Good (NLR ≤ 5 at baseline and ≤5 at 12 weeks), Intermediate (NLR ≤ 5 at baseline and >5 at 12 weeks) and Poor (NLR > 5 at baseline and at 12 weeks).

We then investigated if a change in NLR during treatment affected overall survival. At 12 weeks, NLR ≤ 5 was associated with higher OS. Patients with NLR ≤ 5 had a longer median survival (15.0 months, 95% CI 12.7–21.4), compared to patients with NLR > 5 after 12 weeks (9.5 months, 95% CI 8.3–18.4), *p* = 0.03 (Figure 1b). Patients who had a NLR ≤ 5 at baseline and maintained a NLR < 5 after 12 weeks showed a significantly (*p* = 0.001) better OS with a median of 16.0 months (95% CI 14.8–25.5) compared with a NLR that changed from ≤5 to >5 at 12 weeks, (median OS: 9.1 months, 95% CI 7.1–NR). OS was similarly low in patients who had NLR >5 at baseline and at 12 weeks ((median 8.7 months, 95% CI 8.3–NR) and patients whose NLR changed from ≤5 to >5 at 12 weeks (Table 3 and Figure 2). When investigating absolute change of the NLR, we found that the higher the increase in NLR between baseline and 12 weeks the larger the hazard ratio (Supplementary Figure S1).

#### 3.2.3. Evolution of ANC and ALC during Therapy

In general, during therapy the ANC and ALC tended to decrease as the treatment cycles progressed (Supplementary Figure S2).

### 3.3. Primary Outcome within Docetaxel Treated Population

#### 3.3.1. Association of NLR at Baseline with Overall Survival

**Figure 3:**
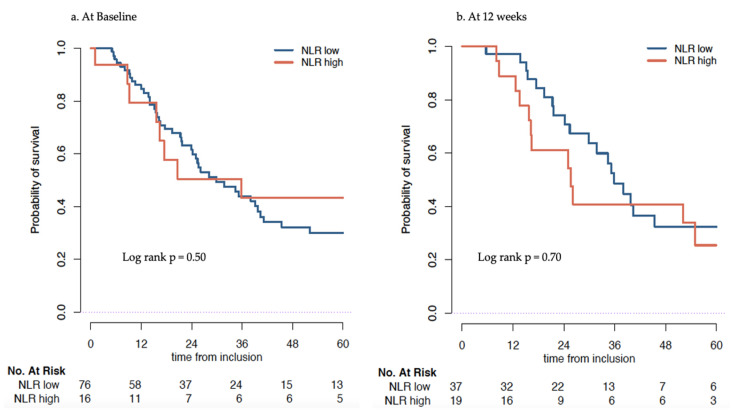
Overall survival (months) of patients treated with Docetaxel estimated by Kaplan–Meier stratified by Neutrophil to Lymphocyte ratio (NLR) low (≤5) and NLR high (>5) calculated at baseline (**a**) and at 12 weeks (**b**).

In the docetaxel cohort, the median OS was 29.9 (24.2–41.1) months at baseline for NLR ≤ 5 patients and 35.8 (16.4-Not Reached (NR)) months for patients with a NLR > 5. No association was found between the NLR and overall survival (*p* = 0.50) Likewise, after 12 weeks of docetaxel, the median OS was 35.8 (29.9–NR) months for low NLR and 25.6 (16.2–NR) months for high NLR. In multivariable analysis, a low NLR was not significantly associated with a better OS, *p* = 0.51 (Table 4, Figure 3 and Supplementary Table S2). 

#### 3.3.2. Association of Change of NLR at 12 Weeks with OS

**Figure 4:**
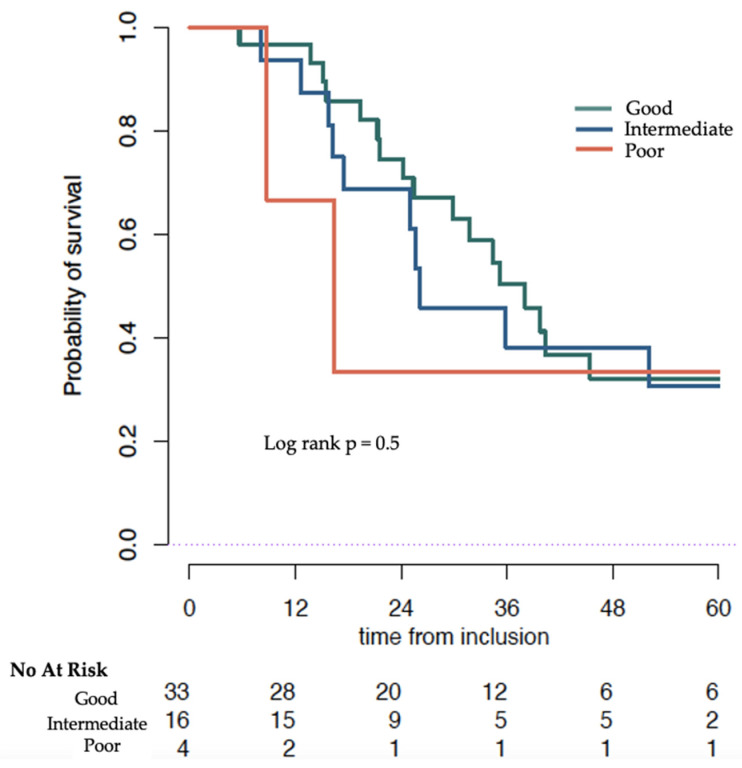
Overall survival (months) of patients treated with Docetaxel estimated by Kaplan–Meier stratified by Neutrophil to Lymphocyte ratio (NLR) score: Good (NLR ≤ 5 at baseline and ≤5 at 12 weeks), Intermediate (NLR ≤ 5 at baseline and >5 at 12 weeks) and Poor (NLR > 5 at baseline and at 12 weeks).

Patients who had a NLR ≤ 5 at baseline and maintained a NLR < 5 at 12 weeks had a median OS of 38.1 months (95% CI 30.0–NR). If the NLR changed from ≤ 5 to > 5 at 12 weeks, median OS was 26.1 months, 95% CI 17.1–NR). In patients with NLR > 5 at baseline whose NLR remained >5 at 12 weeks median OS was 16.4 months, 95% CI 8.8–NR. We were unable to find a significant difference in OS when the NLR changed from <5 to >5 during treatment (*p* = 0.86) (Table 5 and Figure 4).

#### 3.3.3. ANC and ALC Evolution during Therapy

During therapy, unlike patient treated by ^223^Ra, the ANC and ALC tended to remain stable as treatment progressed (Supplementary Figure S3).

## 4. Discussion

In this study, we found that a low NLR at baseline as well as at 12 weeks after the start of treatment was associated with better overall survival in patients treated with ^223^Ra but not in patients treated with docetaxel. Patients with a baseline NLR ≤ 5 as well as low (≤5) NLR at 12 weeks of treatment had a significantly longer median survival (16.0 months) than patients whose NLR was low (≤5) at baseline and increased to >5 at 12 weeks (9.1 months). Regarding overall survival, in our study, ^223^Ra-treated patients with a baseline NLR ≤ 5 had 14.5 months (95% CI 10.2–16.4) median overall survival. These results are consistent with the phase 3 ALSYMPCA study in which patients treated with ^223^Ra had a median overall survival of 14 months [11]. 

The fact that a high NLR at baseline is associated with poor OS in many metastatic cancers is well documented [5,6,7,8]. In our study, after multivariable analysis, adjusted on prior docetaxel treatment, previous prostatectomy, ECOG, PSA level, Hemoglobin, tALP, a low NLR (≤5) at baseline was associated with a better prognosis in ^223^Ra-treated patients. These results are consistent with the available literature regarding ^223^Ra-treated patients. Indeed, the fact that the baseline NLR is a prognostic factor in ^223^Ra-treated patients is well recognized in most studies on the subject including the post hoc analysis of the ALSYMPCA trial by Meisel et al. [11,15] and other trials [16,17]. Our results are unique in finding that not only baseline NLR but also the change of the NLR at 12 weeks is a predictive factor and could help selecting patients who could benefit from changing their systemic treatment. Lorente et al. found in mCRPC patients treated with cabazitaxel that not only a favorable baseline NLR (<3) but also a conversion to a favorable NLR at 12 weeks was a significant predictive factor of improved OS (HR 0.66, 95% CI 0.51–0.85, *p* = 0.001) [18]. These results are consistent with Lalani et al. who found that in patients treated with multiple regimens of an immune checkpoint blockade for metastatic renal cell cancer a higher NLR at 6 weeks was a significantly stronger predictor of all analyzed outcomes than the baseline value. More precisely, a change of ≥25% from baseline to 6 weeks was associated with worse outcomes compared to a decrease in NLR by ≥25% [9]. The same results were obtained, in a cohort of more than a thousand patients with lung cancer. Mezquita et al. explored the derived neutrophil to leucocyte ratio (dNLR) predictive impact in patients treated with immune checkpoint inhibitors for non-small cell lung cancer. Again, the change in dNLR was prognostic [10]. 

In the docetaxel-treated group, we found that the NLR at baseline and at 12 weeks was not associated with OS. Even if our findings are consistent with Sumbul et al. and Linton et al. [19,20], many other studies have shown, for different NLR cut-offs, that the NLR ratio was associated with longer OS in chemotherapy-treated patients [3,18,21,22,23]. This difference in our patients could be explained by a lack of statistical power and the fact that prostate cancer is considered an immunologically cold cancer [24].

Moreover, we found that during treatment with ^223^Ra the ANC and ALC tended to decrease. This could help explain the mechanism of the relationship between increased NLR and poor OS in ^223^Ra-treated patients. Lymphocytes have been known to play a key role in maintaining tissue homeostasis, and therefore prevent uncontrolled tumor cells growth. Among various cancer defense mechanisms, cytotoxic CD8 T-cells are responsible for mediating cancer cells destruction, while T-regs cells allow for a control of the inflammatory environment [25]. Ionizing radiation agents have been used for years in cancer treatment for their direct cytocidal effect on tumor cells, but they are also known to cause inflammation and modulate the host’s immune response to cancer [25,26]. Several studies have shown the hematological toxicity of ^223^Ra [11,27]. Furthermore, Pike et al. have shown that lymphopenia induced by prolonged extracranial radiotherapy treatment can decrease OS in patients receiving immune checkpoint inhibitors [28]. High-linear energy transfer (LET) particles such as ^223^Ra are commonly used for the treatment of castration-resistant prostate cancer (CRPC) patients with symptomatic bone metastases. It has been shown that alpha-emitting atoms from a ^223^Ra source induce a systemic inflammatory response [29]. This effect could potentially at the same time decrease its efficacy through its lymphotoxic effect, as has been shown in our study. In the ALSYMPCA trial, ^223^Ra caused any grade thrombocytopenia in 12% and neutropenia in 5% of patients [11].

Our study is not without some limitations. Although there was no significant baseline difference for other clinical factors between the low and high NLR groups for each treatment independently, the ^223^Ra-treated patients and docetaxel-treated patients were not comparable. Several patients in the ^223^Ra group had received docetaxel before treatment with ^223^Ra and ^223^Ra was in general given as the last or before-last treatment line. It’s also important to underline the retrospective nature of our study. Thus, we did not have the data on the use of corticoids or hematopoietic growth factor in the chemotherapy group which could change neutrophil count and change the NLR. Finally, we use a cut off of 5 to determine low and high NLR. However, there is no consensus regarding the optimal cut off. The Neutrophil to Lymphocyte ratio remains a low-cost and minimally invasive easily measured biomarker. It can certainly be used as risk stratification for metastatic PCa in clinical trials or daily practice. More research into risk stratification using the NLR variable as a biomarker is needed to find the optimal cut off. 

## 5. Conclusions

The NLR at baseline and at 12 weeks of treatment appears to have prognostic value for patients with metastatic prostate cancer patients treated with ^223^Ra but this was not found to be the case for patients treated with docetaxel. Statistically, significantly better OS was observed in patients with a NLR ≤ 5 both at baseline and at 12 weeks. A high NLR at baseline and at 12 weeks of therapy is associated with poor OS as well as those patients with NLR ≤ 5 at baseline that converted to a NLR > 5 at 12 weeks. With its low-cost and the fact that it is easily measurable, we believe that the change in NLR during treatment with ^223^Ra has the potential as a prognostic biomarker to guide clinical decisions. Further validating studies are needed to confirm these results as well as to assess the factors that influence NLR in metastatic prostate cancer.

## Figures and Tables

**Table 1:** (**a**): Baseline characteristics of patients treated with ^223^Ra tabulated according to NLR. All values are median (IQR) or frequencies (%). (**b**): Baseline characteristics of patients treated with Docetaxel tabulated according to NLR. All values are median (IQR) or frequencies (%).

(**a**)				
	**Whole Sample** **(n = 149) (%)**	**NLR Low (** **≤** **5)** **(n = 105) (%)**	**NLR High (>5)** **(n = 23) (%)**	***p*** **-Value**
Prior Docetaxel treatment				0.21
No	65 (43.6%)	42 (40%)	6 (26.1%)	
Yes	84 (56.4%)	63 (60%)	17 (73.9%)	
Age (years)	72 (65; 79)	71 (64; 77)	74 (66; 79.5)	0.83
Missing	1	1	0	
ECOG				0.04
0	50 (36.8%)	42 (43.8%)	4 (17.4%)	
1	68 (50%)	45 (46.9%)	15 (65.2%)	
2	18 (13.2%)	9 (9.4%)	4 (17.4%)	
Missing	13	9	0	
PSA levels (ng/mL)	53.2 (14.8; 182)	38.3 (10.1; 122.5)	86.2 (23.8; 307.6)	0.79
Missing	7	1	0	
tALP (IU/L)	106 (69.5; 198.5)	96 (67; 156)	86.2 (23.8; 307.6)	0.1
Missing	18	6	0	
Hb (g/L)	122 (113.8; 133)	96 (67; 156)	158 (92; 267)	0.07
Missing	9	0	0	
White blood count (10^9^/L)	6.5 (5.3; 7.9)	6.2 (5.2; 7.9)	7.7 (6.8; 9)	0.01
Neutrophils (10^9^/L)	4.4 (3.5; 5.6)	4.1 (3.3; 5.1)	5.7 (5.3; 6.6)	0.001
Lymphocytes (10^9^/L)	3.5 (2.5; 4.6)	1.4 (1.1; 1.9)	1 (0.7; 1.1)	<0.001
NLR	3.5 (2.5; 4.6)	3.2 (2.3; 4)	5.8 (5.4; 7)	
Missing	21	0	0	
Platelets (10^9^/L)	239 (186.8; 287)	237 (186; 285)	246 (209; 322)	0.16
Missing	9	0	0	
(**b**)				
	**Whole Sample** **(n = 170) (%)**	**NLR Low (** **≤** **5)** **(n = 76) (%)**	**NLR High (>5)** **(n = 16) (%)**	***p*** **-Value**
Age	67 (59; 74)	69 (59.5; 75.5)	66.5 (59.8; 69.2)	0.15
Missing	1	1	0	
Cancer Type				
Hormone-sensitive	20 (12%)	14 (18.7%)	3 (18.8%)	1
Castration resistant	147 (88%)	61 (81.3%)	13 (81.2%)	
Missing	3	1	0	
ECOG				0.25
0	55 (43.7%)	25 (58.1%)	4 (36.4%)	
1	57 (45.2%)	12 (27.9%)	6 (54.5%)	
2	14 (11.1%)	12 (27.9%)	1 (9.1%)	
Missing	44	33	5	
PSA levels (ng/mL)	29.4 (5.8; 115.7)	20.4 (5.6; 94.3)	121.4 (16.6; 567.8)	0.09
Missing	34	0		
tALP (IU/L)	84.5 (63.8; 168.2)	86 (67; 167)	90 (68.8; 292)	0.1
Missing	74	7	0	
Hb (g/L)	130 (119; 143)	132 (120.8; 143.2)	122.5 (111.5; 134)	0.14
Missing	77	0	0	
White blood count (10^9^/L)	6.9 (5.5; 8.0)	6.8 (5.5; 7.8)	7.6 (5.8; 8.7)	0.16
Neutrophils (10^9^/L)	4.2 (3.4; 5.3)	4.0 (3.2; 5)	6.0 (4.6; 6.9)	0.003
Lymphocytes (10^9^/L))	1.5 (1.1; 1.9)	1.6 (1.3; 2)	0.8 (0.7; 1)	<0.001
NLR	2.6 (2.1; 4.2)	2.4 (1.9; 3)	7.9 (5.6; 8; 7)	
Missing	78	0	0	
Platelets (10^9^/L)	227 (183.8; 264)	230 (183; 261)	200 (175.2; 270.8)	0.29
Missing	78	0	0	

Eastern Cooperative Group (ECOG) performance status, prostate specific antigen (PSA) level, hemoglobin level (Hb), total alkaline phosphatase (tALP), and Neutrophil-to-lymphocyte ratio (NLR) was calculated as the ratio of the absolute neutrophil count (ANC) divided by the absolute lymphocyte count (ALC).

**Table 2:** Median overall survival (months) of patients treated with ^223^Ra according to Neutrophil to Lymphocyte ratio (NLR).

	NLR Low (≤5)	NLR High (>5)	HR (95% CI) *	*p*-Value
baseline	14.5 (10.2–16.4)	8.5 (6.8–10.5)	1.7 (1.00–2.90)	0.05
at 12 weeks	15.0 (12.7–21.4)	9.5 (8.3–18.4)	1.88 (1.07–3.29)	0.03

* Hazard Ratio (HR) adjusted on docetaxel treatment, previous prostatectomy, Eastern Cooperative Group (ECOG) performance status, prostate specific antigen (PSA) level, hemoglobin level (Hb), total alkaline phosphatase (tALP).

**Table 3:** Median overall survival of patients treated with ^223^Ra according to NLR score.

	Good	Intermediate	Poor	*p*-Value
MedianOS Months (95% CI)	16.0 (14.8–25.5)	9.1 (7.1–NR)	8.7 (8.3–NR)	Log rank 0.0001
HR (95% CI)	Ref	3.04 (1.62–5.68)	2.88 (1.31–6.32)	Cox model 0.001

Hazard Ratio (HR), Not Reached (NR), Overall Survival (OS), Reference (Ref), Neutrophil to Lymphocyte Rate (NLR) Score: Good: NLR baseline ≤ 5 Furthermore, NLR at 12 week ≤ 5, Intermediate: NLR at baseline ≤ 5 and at 12 week > 5, Poor: NLR at baseline > 5 and NLR at 12 week > 5.

**Table 4:** Median overall survival (months) of patients treated with Docetaxel according to NLR.

	NLR Low (≤5)	NLR High (>5)	HR (95% CI) *	*p*-Value
Baseline	29.9 (24.2–41.1)	35.8 (16.8–NR)	0.78 (0.37–1.6)	0.50
At 12 weeks	35.8 (29.9–NR)	25.6 (16.2–NR)	1.27 (0.62–2.61)	0.51

* Hazard Ratio (HR) adjusted on docetaxel treatment, previous prostatectomy, Eastern Cooperative Group (ECOG) performance status, prostate specific antigen (PSA) level, hemoglobin level (Hb), total alkaline phosphatase (tALP).

**Table 5:** Median overall survival of patients treated with Docetaxel according to NLR score.

	Good	Intermediate	Poor	*p*-Value
MedianOS months (95% CI)	38.1 (30.0–NR)	26.1 (17.4–NR)	16.4 (8.8–NR)	Log rank 0.3
HR (95% CI)	Ref	1.11 (0.51–2.42)	1.49 (0.34–6.47)	Cox model 0.86

Hazard Ratio (HR), Not Reached (NR), Overall Survival (OS), Reference (Ref), Neutrophil to Lymphocyte Rate (NLR), Score: Good: NLR baseline ≤ 5 and NLR at 12 weeks ≤ 5, Intermediate: NLR at baseline < 5 and at 12 weeks > 5, Poor: NLR at baseline > 5 and NLR at 12 weeks > 5.

## Data Availability

Data presented is contained within the article; for additional information, data sets are also available upon request from the corresponding author.

## Supplementary Materials

The following supporting information can be downloaded at: https://www.mdpi.com/article/10.3390/cancers14194606/s1, Table S1: Adjustment variables in multivariate Cox proportional hazards regression model predicting overall survival in Radium-223 treated patients; Table S2: Adjustment variables in multivariate Cox proportional hazards regression model predicting overall survival in Docetaxel treated patients. Figure S1: Relation between absolute change of Neutrophil to lymphocyte ratio and Hazard Ratio for overall survival for Radium-223 treated patients and Docetaxel treated patients. Figure S2: Evolution of absolute neutrophil count and absolute lymphocyte count during therapy with Radium-223 treated patients. Figure S3: Evolution of absolute neutrophil count and absolute lymphocyte count during therapy in docetaxel treated patients.
