# Supplementary material for: Change of the Neutrophil-to-Lymphocyte Ratio during Treatment: A Potential Prognostic Biomarker in Metastatic Prostate Cancer Treated with Radium-223 Dichloride
Source: Cancers (Basel). 2022 Sep 22;14(19):4606. doi: 10.3390/cancers14194606 (PMC9559675; doi:10.3390/cancers14194606)

Supplementary Materials

Table S1: Adjustment variables in multivariate Cox proportional hazards regression model predicting over-all survival in Radium-223 treated patients.

	HR	95%CI	P-Value
Prior Docetaxel treatment	2.09	1.24-3.52	0.005
Previous prostatectomy	0.32	0.07-1.52	0.15
ECOG	2.5	1.14	0.02
PSA levels	1.0	1.0-1.001	0.06
tALP (IU/L)	1	0.99-1.0	0.10
Hb (g/L)	0.96	0.95-0.98	0
NLR >5	1.7	1.0 – 2.90	0.05

Hazard Ratio (HR), Eastern Cooperative Group (ECOG) performance status, prostate specific antigen (PSA) level, hemoglobin level (Hb), total alkaline phosphatase (tALP)

Figure S1: Relation between absolute change of Neutrophil to lymphocyte ratio and Hazard Ratio for overall survival for Radium-223 treated patients and Docetaxel treated patients.

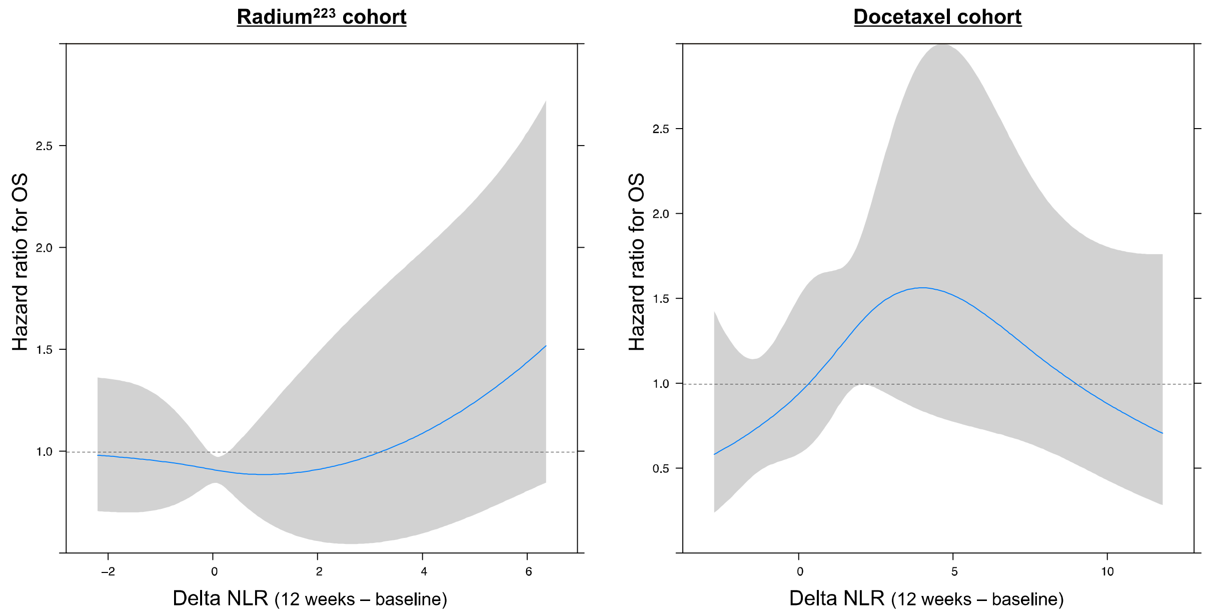

Delta NLR (Neutrophil to lymphocyte ratio) measured as NLR at 12 weeks minus NLR at baseline.

Figure S2: Evolution of absolute neutrophil count and absolute lymphocyte count during therapy with Ra-dium-223 treated patients.

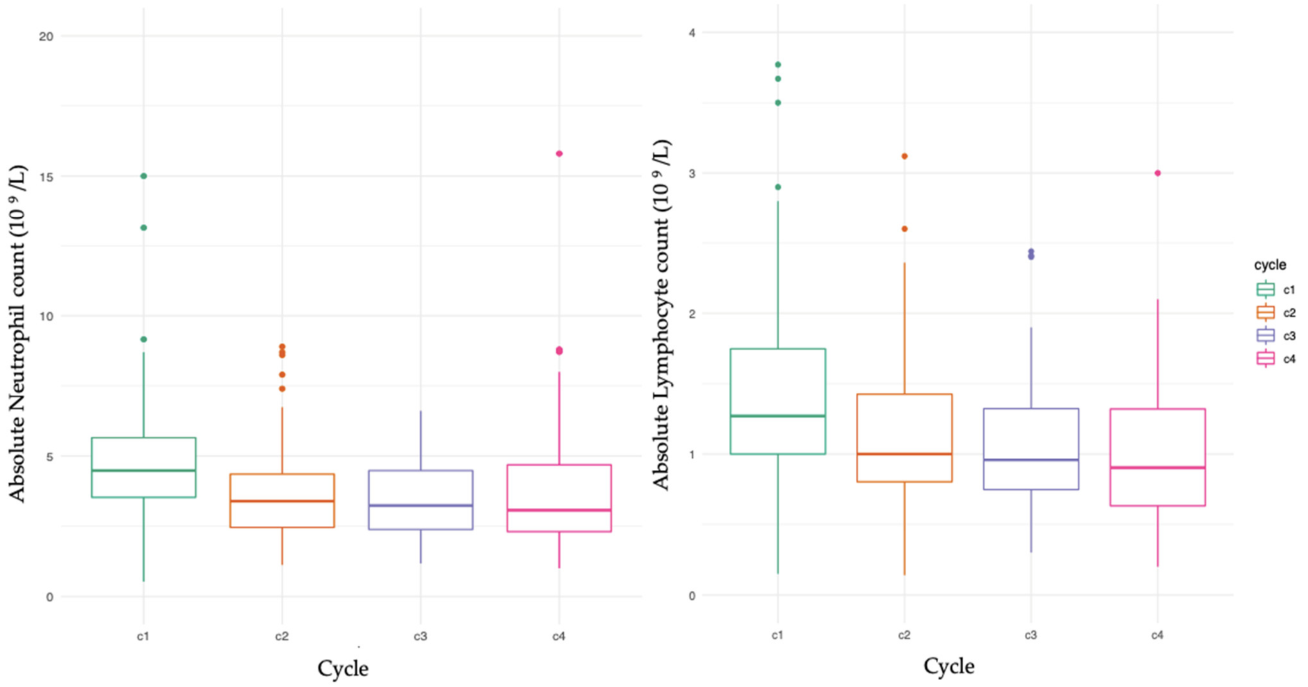

Table S2: Adjustment variables in multivariate Cox proportional hazards regression model predicting over-all survival in Docetaxel treated patients.

	HR	95%CI	P-Value
Age	0.94	0.48-1.83	0.87
Type of Disease	1.27	0.56-2.90	0.55
PSA levels	1.0	1.0-1.002	0.15
tALP (IU/L)	1	0.99-1.0	0.47
Hb (g/L)	0.98	0.96-0.99	0.03
NLR >5	0.78	0.37 -1.6	0.50

Hazard Ratio (HR), Prostate specific antigen (PSA) level, hemoglobin level (Hb), total alkaline phosphatase (tALP), and Neutrophil-to-lymphocyte ratio (NLR) was calculated as the ratio of the absolute neutrophil count (ANC) divided by the absolute lymphocyte count (ALC).

Figure S3: Evolution of absolute neutrophil count and absolute lymphocyte count during therapy in docet-axel treated patients.

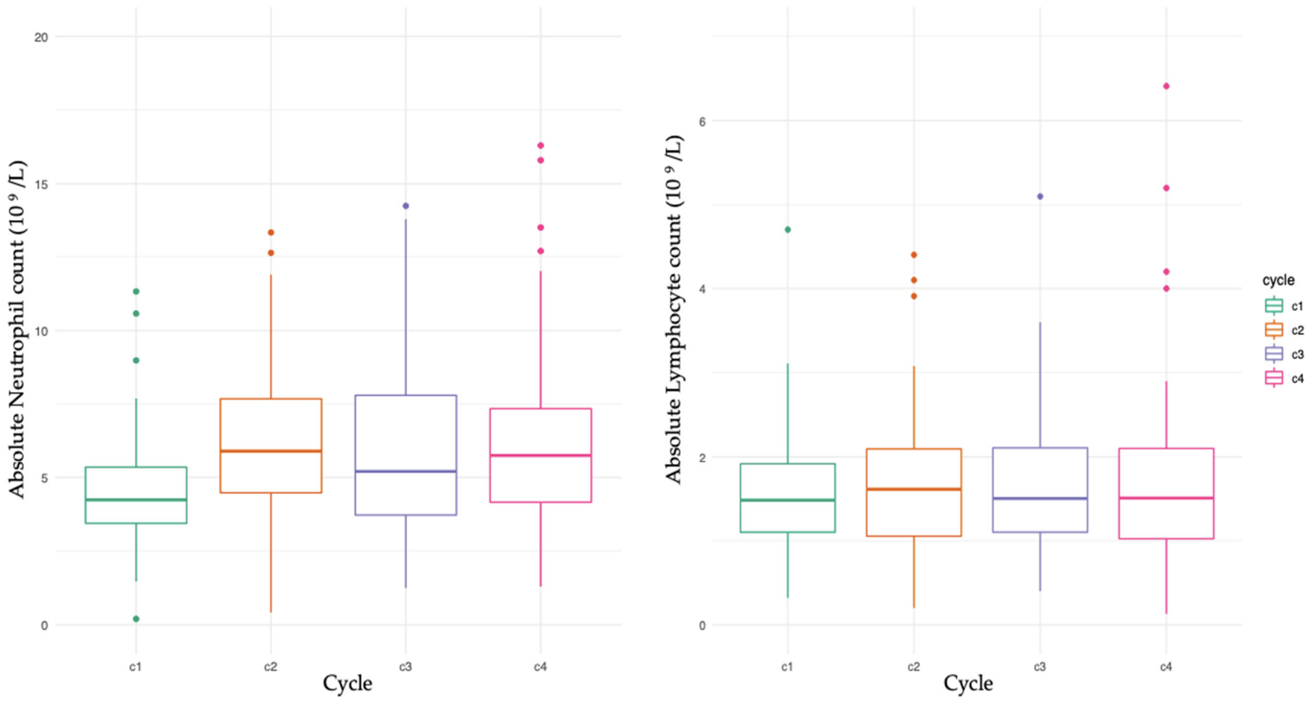

Supplement: Supplementary file 1 [file cancers-14-04606-s001.zip › cancers-1905200-supplementary.pdf]
